# Interaction of Polyelectrolytes with Proteins: Quantifying the Role of Water

**DOI:** 10.1002/advs.202100661

**Published:** 2021-05-03

**Authors:** Jacek J. Walkowiak, Matthias Ballauff

**Affiliations:** ^1^ Institut für Chemie und Biochemie Freie Universität Berlin Taktstraße 3 Berlin 14195 Germany; ^2^ Aachen‐Maastricht Institute for Biobased Materials Maastricht University Brightlands Chemelot Campus, Urmonderbaan 22 Geleen 6167 RD The Netherlands

**Keywords:** complex formation, counterion release, hydrophobic interaction, polyelectrolyte, proteins

## Abstract

A theoretical model is presented for the free energy Δ*G*_b_ of complex formation between a highly charged polyelectrolyte and a protein. The model introduced here comprises both the effect of released counterions and the uptake or release of water molecules during complex formation. The resulting expression for Δ*G*_b_ is hence capable of describing the dependence of Δ*G*_b_ on temperature as well as on the concentration of salt in the system: An increase of the salt concentration in the solution increases the activity of the ions and counterion release becomes less effective for binding. On the other hand, an increased salt concentration leads to the decrease of the activity of water in bulk. Hence, release of water molecules during complex formation will be more advantageous and lead to an increase of the magnitude of Δ*G*_b_ and the binding constant. It is furthermore demonstrated that the release or uptake of water molecules is the origin of the marked enthalpy–entropy cancellation observed during complex formation of polyelectrolytes with proteins. The comparison with experimental data on complex formation between a synthetic (sulfated dendritic polyglycerol) and natural polyelectrolytes (DNA; heparin) with proteins shows full agreement with theory.

## Graphical abstract

Schematic illustration of protein and polyelectrolyte is presented. Binding is accompanied by release of counterions and water molecules.

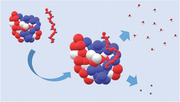

## 1 Introduction

Charge–charge interaction is of central importance for the formation of a complex between a polyelectrolyte and a protein.^[^ ^1^ ^]^ Thus, highly charged polyelectrolytes as, e.g., DNA or heparin are ubiquitous in nature and understanding their interaction with proteins is of fundamental importance. For DNA being the best‐studied natural polyelectrolyte, this fact was already established in the 1970s of the last century^[^ ^2^ ^]^ and the review by Record et al. marks the progress made in this early work.^[^ ^3^ ^]^ Since this time the interaction of DNA with various proteins has been studied with great care by a number of groups.^[^ ^4^, ^5^, ^6^, ^7^, ^8^, ^9^, ^10^, ^11^, ^12^ ^]^ Interaction of synthetic polyelectrolytes with proteins has been a field of active research since a long time^[^ ^13^, ^14^, ^15^, ^16^, ^17^, ^18^ ^]^ and a review of this work was given recently.^[^ ^19^ ^]^ Counterion release was established as the main mechanism operative in binding:^[^ ^3^ ^]^ The highly charged polyelectrolytes carry condensed counterions that are firmly bound and which do not contribute to the osmotic pressure within the system. For a negatively charged polyelectrolyte as, e.g., DNA the patches of positive charge on the surface of the protein become the multivalent counterion upon complex formation. A concomitant number of condensed counterions is released in this process. The gain of free energy thus effected is purely entropic. Up to now, the binding of DNA to various proteins is by far the best‐studied case.^[^ ^3^, ^12^, ^20^, ^21^ ^]^ However, the scheme is far more general and counterions release is important for the interaction of proteins to a wide variety of highly charged natural polyelectrolytes such as RNA, or highly charged glycoaminoglycans as, e.g., heparin.^[^ ^19^ ^]^

The role of water in complex formation has been discussed early on^[^ ^3^ ^]^ using the theory of linked equilibria^[^ ^22^ ^]^ and binding polynomials.^[^ ^22^, ^23^, ^24^, ^25^ ^]^ Already in 1969 Tanford called attention on the fact that the activity of water is bound to the activity of salt by virtue of the Gibbs–Duhem relation.^[^ ^23^ ^]^ The release or binding of counterions can be accompanied by a release or binding of water molecules the number of which being defined as Δ*w*. This effect leads to an additional term in the expression for the binding free energy scaling as *(m*_s_/*m*_w_)Δ*w* where *m*_s_ denotes the molality of salt ions whereas *m*_w_ is the molality of water (55.6).^[^ ^3^, ^23^ ^]^ Tanford argued that this term is small for salt concentrations not too high. However, for salt concentrations in excess of 0.5 m, the release water will become important and modify the free energy of binding as shown for DNA interacting with natural proteins.^[^ ^7^, ^8^, ^10^, ^11^, ^26^, ^27^ ^]^ In a series of precise and comprehensive experiments, Bergqvist et al. demonstrated that the release of water can reverse the weakening of the binding by added salt and lead to a nonmonotonous dependence of the thermodynamic binding constant *K*_b_ on the concentration *c*_s_ of the added salt.^[^ ^10^, ^11^, ^26^, ^27^ ^]^ A refined discussion of the release of water was given by Record and co‐workers^[^ ^28^, ^29^ ^]^ who suggested that Δ*w* is intimately related to the preferential adsorption of the ions to the surface of the biomolecule (cf. ref. [28] and further references given there). The model of vander Meulen et al.^[^ ^29^ ^]^ predicts that Δ*w* is vanishing if there is no preferential adsorption of the co‐ or counterions. The analysis of experimental data of the binding of proteins to DNA led vander Meulen et al. to the conclusion that Δ*w* is therefore small if salts in the middle of the Hofmeister series^[^ ^30^ ^]^ as, e.g., NaCl or KCl are used. Moreover, the discussion by vander Meulen et al. clearly demonstrates that Δ*w* is not simply the number of released water molecules but the part of the free energy related to the release or uptake of water.

The overview of these studies reveals that the understanding of the influence of various salt ions onto the complex formation between polyelectrolytes and proteins is rather advanced. The influence of temperature, on the other hand, is not so clear yet. Many experimental studies on natural polyelectrolytes as DNA^[^ ^7^, ^8^, ^9^, ^31^, ^32^, ^33^, ^34^, ^35^ ^]^ and synthetic polyelectrolytes^[^ ^36^, ^37^, ^38^, ^39^, ^40^, ^41^ ^]^ demonstrate that the free energy of binding Δ*G*_b_ exhibits either a maximum or a minimum. Hence, the entropy of binding Δ*S*_b_ is very small around the extremum of Δ*G*_b_ and the specific heat Δ*c*_p_ may become much larger than Δ*S*_b_.^[^ ^31^, ^33^, ^34^ ^]^ Thus, early thermodynamic studies already observed that complex formation of polyelectrolytes with proteins is accompanied by a large negative change of the specific heat Δ*c*_p_.^[^ ^42^ ^]^ Similar observations have been made since a long time^[^ ^43^ ^]^ for the unfolding of proteins in aqueous solution. A necessary consequence of a large value of Δ*c*_p_ is the strong compensation of the enthalpy Δ*H*_b_ and entropy Δ*S*_b_ of binding:^[^ ^31^ ^]^ The specific heat Δ*c*_p_ may become much larger than Δ*S*_b_ which in consequence leads to a free energy of binding Δ*G*_b_ that hardly changes with temperature.^[^ ^31^, ^43^ ^]^ The respective enthalpy of binding Δ*H*_b_ and entropy Δ*S*_b_ of binding, on the other hand, must vary strongly with temperature because of the large Δ*c*_p_. This enthalpy–entropy compensation (EEC)^[^ ^31^, ^33^, ^34^, ^44^, ^45^, ^46^ ^]^ has been a subject of intense discussion since the first paper by Lumry.^[^ ^44^ ^]^ However, the EEC is a clearly established fact as shown for biological systems by Jen‐Jacobson and co‐workers.^[^ ^33^, ^34^, ^47^, ^48^ ^]^ Moreover, the EEC has been shown to be an established fact for synthetic systems by Liu and Guo.^[^ ^49^ ^]^ Recently, Schönbeck and coworkers presented a thorough study of the EEC on the interaction of cyclodextrin with guest molecules.^[^ ^50^ ^]^ All experimental studies done so far clearly reveal that the EEC is a general phenomenon which can be observed for a wide variety of synthetic and biological systems and which is related to the change of water networks during complex formation (see also the discussion in refs. [51] and [52]). Up to now, however, there is no quantitative study between the release of water molecules as embodied in Δ*w* and the EEC.

Here we present a general thermodynamic model of the dependence of Δ*G*_b_ on temperature and salt concentration. No specific assumptions are needed for this treatment. The model developed here treats the free energy of binding Δ*G*_b_ in the vicinity of its extremum. Hence, the respective enthalpy of binding Δ*H*_b_ and entropy Δ*S*_b_ of binding vary strongly in this regime and complex formation and the dependence on temperature is governed by a large change of the specific heat Δ*c*_p_. In this regime, Δ*H*_b_ and *T*Δ*S*_b_ can be expanded linearly in temperature^[^ ^31^, ^33^, ^34^ ^]^ which provides a firm basis for the understanding of Δ*G*_b_ as the function of *T*. Moreover, the effect of water uptake or release embodied in the term Δ*w* scales linearly with salt concentration.^[^ ^3^, ^27^, ^29^, ^53^ ^]^ Both facts can be combined to develop a model that comprises the dependence of Δ*G*_b_ on both temperature *T* and salt concentration *c*_s_. The discussion given below demonstrates that effect of both variables is closely interrelated and meaningful studies must hence vary both *T* and *c*_s_. The model presented here can therefore predict the strong EEC seen in these systems. A comparison of theory and experiment, however, requires data of the free energy of binding Δ*G*_b_ over a considerable range of temperatures and salt concentrations that are available only for a few systems. Here we shall reanalyze the data obtained from recent studies of the interaction of lysozyme with heparin^[^ ^54^ ^]^ and with dendritic polyglycerol sulfate.^[^ ^38^ ^]^ Moreover, data stemming from a comprehensive study of the interaction of DNA with a polymerase by LiCata and coworkers^[^ ^7^, ^8^ ^]^ will be compared to theory.

## 2 Theory

We consider the equilibrium of a polyelectrolyte PE with a protein P in aqueous solution in the presence of monovalent salt ions with concentration *c*_s_. The polyelectrolyte is highly charged and a certain fraction of the counterions is therefore condensed to the polyelectrolyte.^[^ ^3^, ^20^, ^55^ ^]^ As in previous work,^[^ ^1^ ^]^ this fraction with be estimated by Manning's theory:^[^ ^55^ ^]^ If *b* is the distance between two charges along the linear polyelectrolyte, a charge parameter *ξ* can be defined through *ξ* = *λ*_B_/*b* where *λ*_B_ is the Bjerrum length (*λ*_B_ = *k*_B_ *T*/4*πɛɛ*_0_ ; *ɛ*: dielectric constant of the medium, *k*_B_: Boltzmann constant, *ɛ*_0_: permittivity of the vacuum, *T* : absolute temperature). If *ξ* > 1, a fraction 1–1/*ξ* of the counterions will be condensed to the linear chain.^[^ ^55^ ^]^ From these considerations a local concentration of condensed counterions *c*_ci_ can be defined^[^ ^20^ ^]^ that is typical of the order of one mol per liter.^[^ ^36^, ^52^, ^56^ ^]^ These ions must be treated as a reaction partner and considered in the stoichiometry of the reaction.^[^ ^3^, ^20^ ^]^ The counterions balancing the charge of the macroion are therefore divided into a condensed part and a part interacting with the macroion via a screened Debye–Hückel interaction. In addition, hydration of the three components may change during complex formation and water (W) must be considered in the stoichiometry of the reaction as well. Hence, for a reaction of a protein *P* with an anionic polyelectrolyte PE to a complex PEP we have^[^ ^3^ ^]^

(1) $$P+\mathrm{PE}\;⇆\mathrm{PEP}+\Delta n_{\mathrm{ci}}M^{+}+\Delta n_{w}\;W$$

whereby Δ*n*_ci_ cations of type *M^+^* condensed to the polyelectrolyte have been released. It should be noted that anions may be released or taken up during binding as well. Therefore, Δ*n*_ci_ denotes the net number of released ions. The quantity Δ*n*_w_ is the net change of bound water molecules during binding.

In the following, we formulate the measured equilibrium constant *K*_b_ in terms of the concentrations of the reactants

(2) $$K_{b}=\frac{\left[\mathrm{PEP}\right]}{\left[P\right]\left[\mathrm{PE}\right]}\;$$

Then it can be shown that^[^ ^3^ ^]^

(3) $$d\ln\;K_{b}=-\left(\Delta n_{\mathrm{ci}}-\frac{pm}{55.6}\Delta w\right)d\ln a_{\pm }+d\frac{\gamma_{\mathrm{PEP}}}{\gamma_{P}\gamma_{\mathrm{PE}}}$$

Here *a*_±_ denotes the mean activity of the added salt, *p* = 2 for monovalent salt with molality *m*. The first term on the right‐hand side is related to the net release/uptake of ions Δ*n*_ci_ whereas the release/uptake of water in the course of complex formation is treated in terms of the parameter Δ*w* independent of salt concentration. Water does not constitute an independent component, however, but its activity is bound to the activity of the salt ions by virtue of the Gibbs–Duhem relation.^[^ ^23^ ^]^ The second term on the right‐hand side contains the ratio of the activity coefficients *γ*_PEP_, *γ*_P_, and *γ*_PE_ of the complex, the protein, and the polyelectrolyte, respectively. These activity coefficients are related to the Debye–Hückel interactions of the reaction partners. As shown by Record et al., a general analysis of the activity coefficients of rodlike polyelectrolytes and their complexes with proteins demonstrates that the term $\ln\frac{\gamma_{P}\gamma_{\mathrm{PE}}}{\gamma_{\mathrm{PEP}}}$ gives a small but non‐negligible contribution to *K*_b_.^[^ ^2^, ^3^ ^]^ For the spherical macroions the term $\ln\frac{\gamma_{P}\gamma_{\mathrm{PE}}}{\gamma_{\mathrm{PEP}}}$ was considered recently and shown to be negligibly in first approximation.^[^ ^52^ ^]^ Given these approximations it is expedient to replace the activity *a*_±_ by the concentration [M^+^] of the monovalent ions. For the dilute solutions of the polyelectrolyte and protein under consideration here, [M^+^] may safely expressed through the concentration of added salt *c*_s_. Since experiments analyzing Δ*w* are often done at high salt concentrations, however, the replacement of replace the activity *a*_±_ by *c*_s_ may become a stringent assumption and possible errors thus incurred must be kept in mind. Within the present approximations the molality *m* can be replaced by *c*_s_.

Given the various assumptions and approximations, Equation (3) may now be rendered for monovalent salt ions as

(4) $$\frac{\mathrm{dln}K_{b}}{\mathrm{dln}c_{s}}=-\Delta n_{\mathrm{ci}}+\frac{2}{55.6}m\Delta w\approx -\Delta n_{\mathrm{ci}}+0.036\Delta wc_{s}$$

Integration of Equation (3) leads to^[^ ^29^, ^53^ ^]^

(5) $$\ln K_{b}-\ln K_{b}\;\left(c_{\mathrm{ref}}\right)=-\Delta n_{\mathrm{ci}}\ln\frac{c_{s}}{c_{\mathrm{ref}}}+0.036\Delta w\left(c_{s}-c_{\mathrm{ref}}\right)$$

Equation (5) is the starting point of the analysis of *K*_b_ by theory. For counterion release embodied in the first term on the right hand side of Equation (5), the reference concentration is given by *c*_ci_, the concentration of condensed counterions on the polyelectrolyte. This term describes hence the change of *K*_b_ when going from a salt concentration *c*_ci_ to a salt concentration *c*_s_. The second term describes the concomitant change of free energy caused by the possible uptake or release of water molecules. Equations (3) and (5) show that Δ*w* is a free energy that will depend on temperature but not on the salt concentration *c*_s_. This quantity hence defies direct interpretation in terms of the number of released water molecules.

From Equation (5) we see that

(6) $$\ln\;K_{b}=-\Delta n_{\mathrm{ci}}\ln c_{s}+0.036\Delta wc_{s}+C$$

where the constant *C* can only depend on temperature but not on salt concentration. The binding constant *K*_b_ can be measured precisely by isothermal titration calorimetry (ITC^[^ ^57^, ^58^ ^]^) and leads to the free energy of binding through

(7) $$\Delta G_{b}=-RT\ln K_{b}$$

Therefore

(8) $$\Delta G_{b}\;\left(T,c_{s}\right)=\;\mathrm{RT}\Delta n_{\mathrm{ci}}\ln c_{s}-\mathrm{RT}0.036\Delta wc_{s}+\Delta G_{\mathrm{res}}$$

The reference free energy Δ*G*_res_ = − *RT* · *C* is independent of salt concentration by virtue of Equation (6).

The derivation of Equations (4)–(8) contains a number of stringent conditions and mostly disregard activity coefficients. However, in view of the limited accuracy of the experimental data, an analysis that carries along the activity coefficients would hardly be possible. Moreover, Equations (4) and (5) have been used repeatedly for the analysis of mainly the complex formation of proteins with various proteins.^[^ ^10^, ^11^, ^26^, ^27^, ^29^, ^35^, ^53^ ^]^ It is hence useful to use this approximation for a comprehensive comparison of theory and experiment. In this way, Equations (4) and (5) can be regarded as the relations that define a model of protein/polyelectrolyte interaction. It is in this sense that Equation (5) is used and analyzed in the following.

Equation (8) demonstrates that *K*_b_ depends on two decisive variables: i) the temperature *T* and ii) the salt concentration *c*_s_ in solution. A comprehensive discussion of the complex formation must therefore comprise a set of experiments in which both variables are changed over the widest range possible. A suitable model must describe the dependence on both *T* and *c*_s_ and contain mixed derivatives with regard to both quantities.

In practically all systems in which polyelectrolytes form complexes with proteins, a large specific heat Δ*c*_p_ is found. For many systems in which DNA is interaction with various proteins, Δ*c*_p_ is found to be negative^[^ ^42^ ^]^ whereas a positive Δ*c*_p_ is detected for several well‐studied synthetic polyelectrolytes.^[^ ^37^, ^38^ ^]^ In these systems, |Δ*c*_p_| ≫ |Δ*S*_b_| and both Δ*H*_b_ and Δ*S*_b_ exhibit a strong variation with temperature. This feature is a direct consequence of the fact that the free energy of binding Δ*G*_b_ exhibits either a maximum or a minimum in the experimental range of temperatures where Δ*S*_b_ *= *0. Hence, two characteristic temperatures must be defined: The entropy Δ*S*_b_ is zero at temperature *T*_s_ whereas Δ*H*_b_ = 0 at a temperature *T*_h_.^[^ ^31^, ^43^ ^]^ Here, *T*_s_ is the temperature where Δ*G*_b_ exhibits an extremum while *T*_h_ is the temperature where the binding constant *K*_b_ has a maximum or a minimum.^[^ ^31^, ^33^, ^34^ ^]^ This extremum of Δ*G*_b_ and the large value of the specific heat are the thermodynamic origin of the EEC.^[^ ^31^ ^]^ Therefore

(9) $$\Delta H_{b}\left(T\right)≅\Delta H_{b}\left(T_{s}\right)+\Delta c_{p}\left(T-T_{s}\right)$$

and

(10) $$\Delta S_{b}≅\Delta c_{p}\ln\frac{T}{T_{s}}$$

Combination of both expressions leads to the well‐known generalized van't Hoff expression^[^ ^8^, ^38^, ^45^, ^52^, ^59^ ^]^

(11) $$\begin{array}{c} \Delta G_{b}=\Delta H_{b,\mathrm{ref}}-T\Delta S_{b,\mathrm{ref}}+\Delta c_{p}\left\lfloor \left(T-T_{\mathrm{ref}}\right)-T\;\ln\left(\frac{T}{T_{\mathrm{ref}}}\right)\right\rfloor \end{array}$$

If the reference temperature *T*_ref_ is set equal to *T*_s_ we obtain

(12) $$\Delta G_{b}=\Delta H_{b}\left(T_{s}\right)+\Delta c_{p}\left\lfloor T-T_{s}-T\;\ln\left(\frac{T}{T_{s}}\right)\right\rfloor$$

Equation (12) demonstrates that the free energy of binding Δ*G*_b_ is dominated by the specific heat Δ*c*_p_ and the temperature of its maximum/minimum *T*_s_. In our previous analysis, both quantities have been found to depend strongly on *c*_s_.^[^ ^38^, ^54^ ^]^ However, the determination of Δ*c*_p_ from experimental data by application of Equation (12) turned out to be difficult and small errors will lead to a marked uncertainty of this quantity and to concomitant errors in Δ*H*_b_ and Δ*S*_b_.

We now turn to a discussion of dependence of Δ*G*_b_ on *c*_s_. We again start with Equation (5) and differentiate with regard to *T*. The dependence Δ*G*_b_ on Δ*n*_ci_ is an entirely entropic effect und thus independent of *T*.^[^ ^29^, ^52^ ^]^ This assumption is in full accord with experimental data on DNA^[^ ^12^ ^]^ and synthetic polyelectrolytes.^[^ ^38^, ^52^ ^]^ Because of ${\left(\partial \ln K_{b}/\partial T\right)}_{c_{s}}=\;\Delta H_{b}/RT^{2}$ we get through differentiation of Equation (5)

(13) $$\frac{1}{RT^{2}}\;\left[\Delta H_{b}\left(c_{s}\right)-\Delta H_{b}\left(c_{\mathrm{ref}}\right)\right]=0.036\left(c_{s}-c_{\mathrm{ref}}\right)\frac{d\Delta w}{dT}$$

Equation (13) shows that Δ*H*_b_(*c*_s_) scales linearly with salt concentration *c*_s_ while Equation (9) states that Δ*H*_b_(*c*_s_) is also depending linearly on temperature. Therefore Δ*H*_b_ must be given by

(14) $$\Delta H_{b}\left(c_{s}\right)=0.036RT^{2}c_{s}\frac{d\Delta w}{dT}+\;\Delta H_{0}$$

where Δ*H*_0_ is an enthalpic contribution that depends neither on *c*_s_ nor on temperature *T*. Therefore, the first derivative of Δ*H*_b_ with regard to salt concentration *c*_s_ is directly related to dΔ*w*/d*T*. This fact shows directly that Δ*w* cannot be a simple stoichiometric coefficient but is related to the free energy of binding. Since *c*_s_ and *T* are independent variables, we obtain a Maxwell relation

(15) $$\frac{\partial^{2}\Delta H_{b}}{\partial c_{s}\partial T}=\;\;\frac{\partial^{2}\Delta H_{b}}{\partial T\partial c_{s}}=\frac{d\Delta c_{p}}{dc_{s}}$$

As already discussed above, Δ*H*_b_ depends linearly on both *c*_s_ and *T*. In consequence, dΔ*c*_p_/d*c*_s_ must be a constant that does neither depend on *T* nor on *c*_s_. This constant is therefore characteristic for given system polyelectrolyte/protein. Hence, conditions (9), (14), and (15) require that *ΔH*_b_(*c*_s_) must be given by the following expression

(16) $$\Delta H_{b}\;\left(c_{s},T\right)=\Delta H_{0}+\frac{d\Delta c_{p}}{dc_{s}}c_{s}\left(T-T_{0}\right)$$

where *T*_0_ is a characteristic temperature specifying the dependence of Δ*w* on *T*. Combining Equation (14) with (16) we obtain

(17) $$\frac{d\Delta c_{p}}{dc_{s}}c_{s}\;\left(T-T_{0}\right)=0.036RT^{2}c_{s}\frac{d\Delta w}{dT}$$

Thus,

(18) $$\frac{d\Delta w}{dT}=\frac{1}{0.036R}\frac{d\Delta c_{p}}{dc_{s}}\frac{T-T_{0}}{T^{2}}$$

which upon integration yields

(19) $$\Delta w\;=\;\Delta w\left(T\right)-\Delta w\;\left(T_{0}\right)=\frac{\frac{d\Delta c_{p}}{dc_{s}}}{0.036R}\left(\ln\frac{T}{T_{0}}+\frac{T_{0}}{T}-1\right)$$

In a similar fashion, the entropy of binding Δ*S*_b_ = $-\;\frac{\partial G_{b}}{\partial T}$ can be derived from Equation (5) as

(20) $$\begin{array}{c} \Delta S_{b}\;\left(c_{s}\right)=-\Delta n_{\mathrm{ci}}R\cdot \ln\left(c_{s}\right)+0.036R\left(\Delta w+T\frac{d\Delta w}{dT}\right)c_{s}+\;\Delta S_{0} \end{array}$$

where Δ*S*_0_ is a constant that does not depend on *c*_s_ nor on *T*. Combining Equations (16) and (20) with Equation (19) we finally get

(21) $$\begin{array}{ccc} & & \Delta G_{b}\left(T,c_{s}\right)=RT\Delta n_{\mathrm{ci}}\ln c_{s}+\Delta H_{0}\\ & & \;-\;T\Delta S_{0\;}+\;c_{s}\frac{d\Delta c_{p}}{dc_{s}}\left\lfloor T-T_{0}-T\ln\left(\frac{T}{T_{0}}\right)\right\rfloor \end{array}$$

This expression is very similar to Equation (11) except for the first term which is due to counterion release. The free energy of binding comprises the constant enthalpic and the entropic reference contributions Δ*H*_0_ and Δ*S*_0_, respectively, both referring to a new characteristic temperature *T*_0_. The last term in Equation (21) describes the dependence on temperature as in Equation (11). It reflects the effect of Δ*w* which is not simply the number of released water molecules. The characteristic temperature *T*_0_ which replaces *T*_s_ is no more a function of the salt concentration *c*_s_. This in turn means that the effect of counterion release is fully covered by the first term in Equation (21). Moreover, the specific heat can now be expressed as

(22) $$\Delta c_{p}=\;c_{s}\frac{d\Delta c_{p}}{dc_{s}}$$

For the residual free energy Δ*G*_res_ defined by Equation (8) we obtain

(23) $$\Delta G_{\mathrm{res}}=\Delta H_{0}-T_{0}\Delta S_{0}$$

Within the above assumptions and approximations, both Δ*H*_0_ and Δ*S*_0_ are independent of *T* and *c*_s_. Therefore Δ*G*_res_ must be a constant as well (see the discussion in ref. [52]). The present theory only considers effects of counterion release and of the release of water while other possible factors as, e.g., hydrogen bonding are not treated explicitly. Possible contributions due to these effects may thus be embodied in the parameters Δ*H*_0_ and Δ*S*_0_.

The above derivation shows clearly that Δ*w* is the part of the free energy of binding related to the release or uptake of water. It is hence interesting to compare the present treatment with the solute partitioning model (SPM) of Record and coworkers.^[^ ^29^, ^30^ ^]^ The SPM treats the interaction of the ions with the dissolved biomolecule in terms of an ion‐specific interaction related to the Hofmeister series and the nonspecific lowering of the water activity by the salt ions. The protein and the polyelectrolyte are strongly hydrated and a part Δ*B*_H2O_ of this shell of water molecules is released (or taken up) upon complex formation. The ions are now partitioned between the bulk solution and the hydration water on the surface. Thus, the equilibrium between these ions can be described by a partition coefficient $K_{p,+\;}=\;(m_{+}^{\mathrm{loc}}$/$m_{+}^{\mathrm{bulk}}$) for the cations where $m_{+}^{\mathrm{loc}}$ denotes the molality of the cations in the hydrated shell whereas $m_{+}^{\mathrm{bulk}}$ is the respective quantity in bulk. The anions are distributed in the same way characterized by the partition coefficient *K*_*p*,−_. With these definitions, Δ*w* can be expressed as

(24) $$\Delta w\;=\;\frac{1}{2}\left(K_{p,+\;}+\;K_{p,-\;}-2\right)\Delta B_{H2O}$$

with omission of a small correction term.^[^ ^29^ ^]^ For systems in which proteins interact with DNA, Δ*B*_H2O_ is expected to a large negative number (see the discussion in ref. [29]). The integer −2 denotes the purely osmotic effect of water release that increases the free energy of complex formation. This situation is encountered if the partition coefficients are small, that is, if the concentration of ions in the hydrated shell is zero. In this case there will be an appreciable gain of free energy if water is released from this hydrated shell into the bulk water having a lower activity due to the salt ions. The effect of water release may be weakened or even reversed if the salt ions interact noncoulombically with the surface of DNA and protein. Here the ions compete with the hydration water of both components. Full compensation takes place if *K*_p,+_ + *K*_p,−_ = 2 which is to be expected for salt in the middle of the Hofmeister series. Then there is no gain if water is released from the hydrated shell into the bulk phase. For *K*_p,+_ + *K*_p,−_ > 2, the effect of water release is reversed by the effect of ion adsorption and raising of the salt concentration will destabilize the complex. Evidently, the partition coefficients *K*_p_ *_,+_* and *K*_p_ *_,−_* are functions of temperature^[^ ^29^ ^]^ and the quantity Δ*w* must be an explicit function of *T* as shown in the present treatment. A fully quantitative comparison of the SPM with the present treatment, however, is not possible since precise data for the dependence of the partitioning coefficient on temperature would be required.

## 3 Results and Discussion

Equation (21) presents the main result of the present analysis. It provides a closed expression for dependence of Δ*G*_b_ on temperature and salt concentration and leads to characterization of the complex formation by a single set of parameters. The various terms in Equation (21) can now be interpreted as follows: Counterion release is fully covered by the first term in full agreement with a large number of studies done on various polyelectrolyte/protein systems.^[^ ^3^, ^12^, ^21^, ^36^, ^37^, ^38^, ^54^ ^]^ Water release leads to the subsequent terms in which the constants contributions Δ*H*_0_ and Δ*S*_0_ refer to all enthalpic and entropic contributions, respectively, at temperature *T*_0_. The last term in Equation (21) takes care of the dependence of Δ*G*_b_ on temperature because of the release of water molecules. Hence, the model predicts that enthalpic and entropic contributions due to water release cancel exactly at *T*_0_. If dΔ*c*_p_/d*c*_s_ is positive, Δ*w *> 0 and the magnitude of free energy with increase with *c*_s_. This is due to the effect that the activity of bulk water is decreasing with increasing *c*_s_ and it becomes more advantageous to release water molecules into this bulk phase. If dΔ*c*_p_/*dc*_s_ < 0, Δ*w* and Δ*c*_p_ are negative and the release of water molecules will require free energy. Hence, the magnitude of Δ*G*_b_ will become smaller with increasing *c*_s_. In all cases the magnitude of the part of Δ*G*_b_ related to the release of water will be determined by dΔ*c*_p_/d*c*_s_ through Equation (22).

Doubts may be raised whether Δ*c*_p_ can vanish with decreasing salt concentration. However, close inspection of Equation (21) reveals that Δ*c*_p_ *= *0 for systems that are entirely driving by counterion release, that is, for system governed entirely by the first term in (21). In this case the free energy of binding is purely entropic and does not exhibit any dependence on *T*. It should be kept in mind, however, that the present model comprises only two effects leading to complex formation, namely counterion release and uptake/release of water. Other factors that may contribute as, e.g., conformational changes of the protein upon binding^[^ ^34^, ^60^ ^]^ are not included. They will in consequence be reflected in the terms Δ*H*_0_ and Δ*S*_0_ that are treated as adjustable parameters in the present model. Moreover, the present model is fully compatible with an additional term in Equation (22) independent of concentration: $\Delta c_{p}=\;\Delta c_{p,0}+\;c_{s}\frac{d\Delta c_{p}}{dc_{s}}$. Such a term Δ*c*_p,0_ would account for possible effects that do not vanish with salt concentration.

The above thermodynamic model requires precise data of *K*_b_ as the function of the two decisive variables *T* and *c*_s_, preferably measured by ITC for the sake of accuracy. We have presented a number of studies of the interaction of sulfated dendritic polyglycerol^[^ ^56^ ^]^ with lysozyme^[^ ^36^, ^38^, ^52^ ^]^ and with human serum albumin.^[^ ^37^ ^]^ In these studies the binding constant *K*_b_ was determined by ITC as the function of both temperature and the concentration of sodium chloride. Moreover, the interaction of heparin with lysozyme was studied in the same way by ITC. A full analysis along the lines devised here, however, can only be done for the system dPGS/lysozyme^[^ ^38^ ^]^ and heparin/lysozyme^[^ ^54^ ^]^ in which *K*_b_ could be measured over a sufficiently broad range of salt concentrations and temperature. As a third example, we chose the very precise set of data obtained for the complex formation of DNA with the polymerase Klentaq by Datta and LiCata.^[^ ^7^, ^8^ ^]^ Here the binding constant was determined over a wide range of temperatures and salt concentrations. This system is more complicated, however, when compared to the system dPGS/lysozyme^[^ ^38^ ^]^ and heparin/lysozyme,^[^ ^54^ ^]^ because complex formation can be accompanied by conformational changes of the protein or a bending of DNA.^[^ ^60^ ^]^

**Figure 1:**
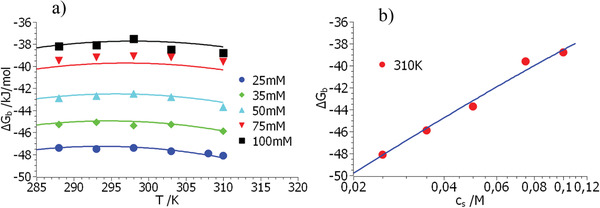
Comparison of the free energy of binding obtained for the system heparin/lysozyme^[^ ^54^ ^]^ with theory (Equation (21)). (a) Displays the measured free energies Δ*G*_b_ as the function of temperature. Parameter is the concentration of salt indicated in the graph. (b) Displays the free energies measured at 310 K as the function of salt concentration. The solid lines in both figures mark the fit by Equation (21) using the parameters in Table 1.

**Figure** **1** displays the experimental data obtained for the system heparin/lysozyme.^[^ ^38^ ^]^ Two features command attention: The dependence of Δ*G*_b_(*T,c*_s_) on temperature is very weak whereas the magnitude of the free energy diminishes strongly with salt concentration *c*_s_. In our previous analysis, both features have been understood in terms of the number of released counterions Δ*n*_ci_ and a temperature *T*_s_ that depends on salt concentration.^[^ ^38^, ^54^ ^]^ No closed expression for Δ*G*_b_(*T,c*_s_) could be offered, however. Equation (11) was used to determine the specific heat Δ*c*_p_ and the enthalpy and entropy of binding for each *c*_s_ as the function of temperature.^[^ ^38^, ^54^ ^]^ The error incurred in the determination of these quantities by use of Equation (11) was considerable.^[^ ^54^ ^]^ Thus, Δ*c*_p_ can only be deduced from Δ*G*_b_(*T,c*_s_) if these data have a minute error only. However, extrapolation of the free energies of binding to a salt concentration of 1 m led for both systems to a constant value for Δ*G*_res_.^[^ ^38^, ^54^ ^]^ This feature was already found by Dragan et al. for a large number of systems in which DNA interacts with various proteins.^[^ ^21^ ^]^ We could demonstrate in a recent paper that Δ*G*_res_ is the free energy resulting at the direct contact between the polyelectrolyte and the protein.^[^ ^52^ ^]^

The comparison of these data with Equation (21) can be done using the *MathLab* routine *cftool* in which the pertinent parameters are fitted at once to the surface defined by the entire set of data Δ*G*_b_(*T,c*_s_). This procedure leads to the parameters gathered in **Table** **1**. Figure 1 displays the comparison of theory and experiment for the system heparin/lysozyme^[^ ^54^ ^]^ in two plots: Figure 1a shows the free energies Δ*G*_b_ measured for different salt concentrations as the function of temperature. Evidently, the slight curvature with *T* is correctly described by theory. Some data are less well described but it is important to note that a single set of parameters (see Table 1) must fit all data at once. Figure 1b displays the measured free energies Δ*G*_b_ measured at 310 K as the function of salt concentration. There is a slight curvature of this semilogarithmic plot that has been used so often to determine Δ*n*_ci_ by a linear fit. Finally, the quantity Δ*G*_res_ defined by Equation (23) follows as −20.8 kJ mol^–1^ whereas the experimental data lead to values between −20 and −22 kJ mol^–1^ (see Table 2 of ref. [54]).

**Table 1:** Summary of the constants characterizing the interaction of proteins with polyelectrolytes according to Equation (21)

System^a)^	*T*_0_ [K]	Δ*n*_ci_	dΔ*c*_p_/d*c*_s_ [kJ mol^–1^ K^–1^ m ^–1^]	Δ*H*_0_ [kJ mol^–1^]	Δ*S*_0_ [kJ mol^–1^ K^–1^]
Heparin/lysozyme^[^ ^54^ ^]^	296	2.95	39.1	−41.70	−0.071
dPGS/lysozyme^[^ ^38^ ^]^	290	2.51	−14.8	−14.75	0.016
DNA/Klentaq^[^ ^7^, ^8^ ^]^	312	3.0	−31.9	−22.30	0.011

^a)^ *T*_0_: characteristic temperature (Equation (16)); Δ*n*_ci_: net number of released counterions (Equation (4)); dΔ*c*_p_/d*c*_s_: coefficient characterizing the dependence of Δ*w* on temperature and salt concentration (Equation (15)); Δ*H*_0_ and Δ*S*_0_: enthalpic and entropic contributions, respectively, at *T = T*_0_ (cf. Equations (14) and (20)).

It may appear doubtful to derive five parameters from the data displayed in Figure 1. However, *T*_0_ can already been read off the position of the maximum or minimum of Δ*G*_b_ whereas Δ*n*_ci_ can be derived by plots of Δ*G*_b_ versus log *c*_s_ (cf. Figure 1b) quite securely. Hence, these parameters have an error of the order of 5% at the most. Δ*H*_0_ and Δ*S*_0_ were found to be rather insensitive toward small errors in *T*_0_ and Δ*n*_ci_ so that the error of these quantities is of the order of 10%. This is in agreement with the observation that Δ*G*_res_ as defined through Equation (23) turns out to be a constant within small limits of errors.^[^ ^21^, ^52^, ^54^ ^]^ The accuracy of the quantity dΔ*c*_p_/d*c*_s_, on the other hand, depends very much on the temperature range in which Δ*G*_b_ was measured. Here the systematic error may exceed 10%. In total, the fit of the parameters is secure since a whole matrix of data on Δ*G*_b_(*T,c*_s_) is fitted by the above procedure.

It is important to note that the plots of Δ*G_b_* against log *c*_s_ may deviate from linearity when the third term in Equation (21) becomes more dominant. A strictly linear behavior has been found for a great number of systems in which DNA interacts with various proteins (cf. the discussion of this point by Privalov et al. in ref. [12]). Notable exceptions of this rule have been found by Dubin et al. for linear polyelectrolytes interacting with proteins at very low ionic strength.^[^ ^14^ ^]^ Here a maximum of log *K*_b_ is found when plotted against log *c*_s_. Unfortunately, no comparison with Equation (21) can be done since the data have been measured only at one temperature. Further measurements on such systems at different temperatures as suggested here must be done to elucidate this point further.

**Figure 2:**
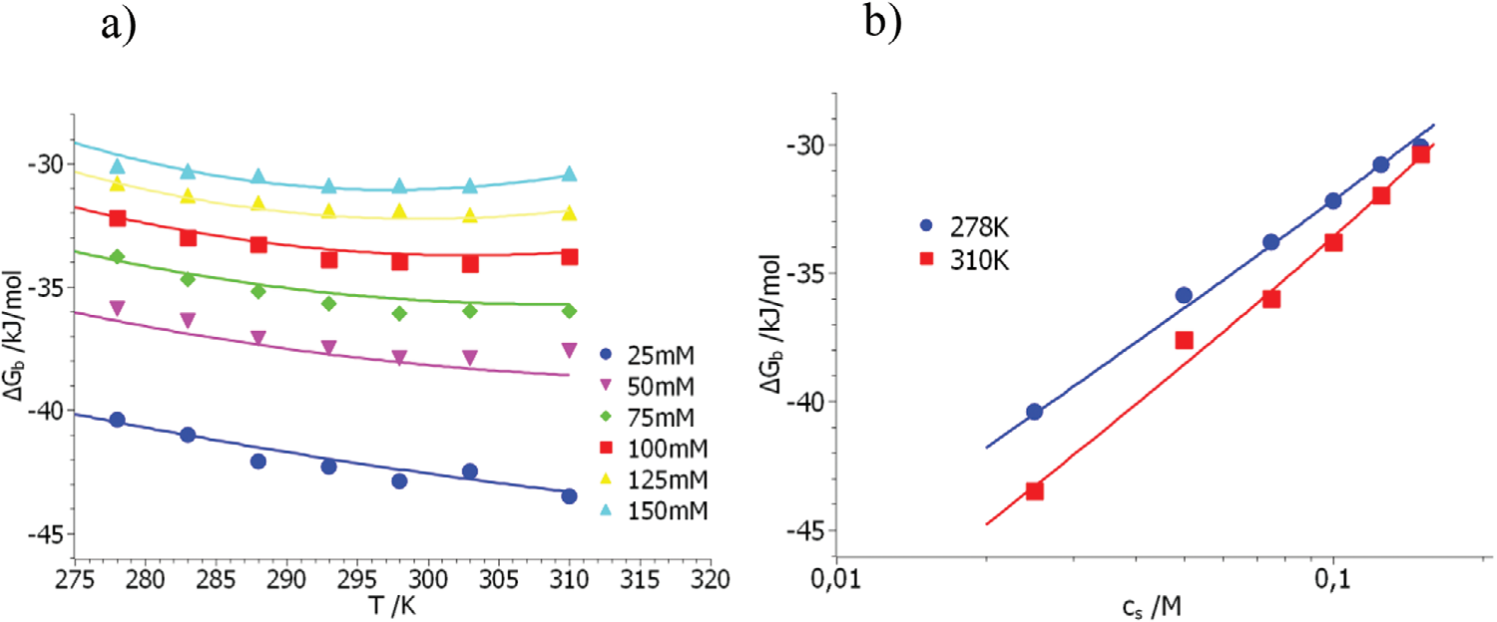
Comparison of the free energy of binding obtained for the system dPGS/lysozyme^[^ ^38^ ^]^ with theory (Equation (21)). (a) Displays the measured free energies Δ*G*_b_ as the function of temperature. Parameter is the concentration of salt indicated in the graph. (b) Displays the free energies measured at 310 K as the function of salt concentration. The solid lines mark the fit by Equation (21) using the parameters in Table 1.

**Figure** **2** displays the set of data obtained for the system dPGS/lysozyme. Again, a single set of parameters (see Table 1) leads to a very satisfactory fit of the data, all measured free energies agree with theory within the limits of error as shown in Figure 2a. The general features of the model are clearly visible: The lowermost curve in Figure 2a displays the data obtained at the lowest salt concentration. Here the measured free energies decrease in a nearly linear fashion because Δ*G*_b_(*T,c*_s_) is dominated by the first term in Equation (21). The last term describing the dependence on temperature is small and nearly negligible. Increasing the concentration *c*_s_ of added salt by a factor of 6 (uppermost curve in Figure 2a) leads to a marked weakening of counterion release. At the same time, the magnitude of the last term in Equation (21) is increased by a factor of 6 and governs the dependence of Δ*G*_b_(*T,c*_s_) on temperature. A second point in Figure 2 commands attention: The free energies as the function of salt concentration *c*_s_ measured at 310 K shown in Figure 2b exhibit a slight curvature whereas the data taken at 278 K are linear within the limits of experimental error. This is due to the fact that 310 K is far above the characteristic temperature of 290 K and the hydration terms in Equation (21) start to play a more important role when *c*_s_ is increasing. Evidently, the conventional fit by a straight line used so often cannot not be entirely correct. Finally, the quantity Δ*G*_res_ as calculated from the parameters in Table 1 through Equation (23) follows as −19.4 kJ mol^–1^. From the experimental data we previously found a value of −20.3 kJ mol^–1^ (see the discussion in ref. [52]). Given the various sources of error, this may be regarded as good agreement.

**Figure 3:**
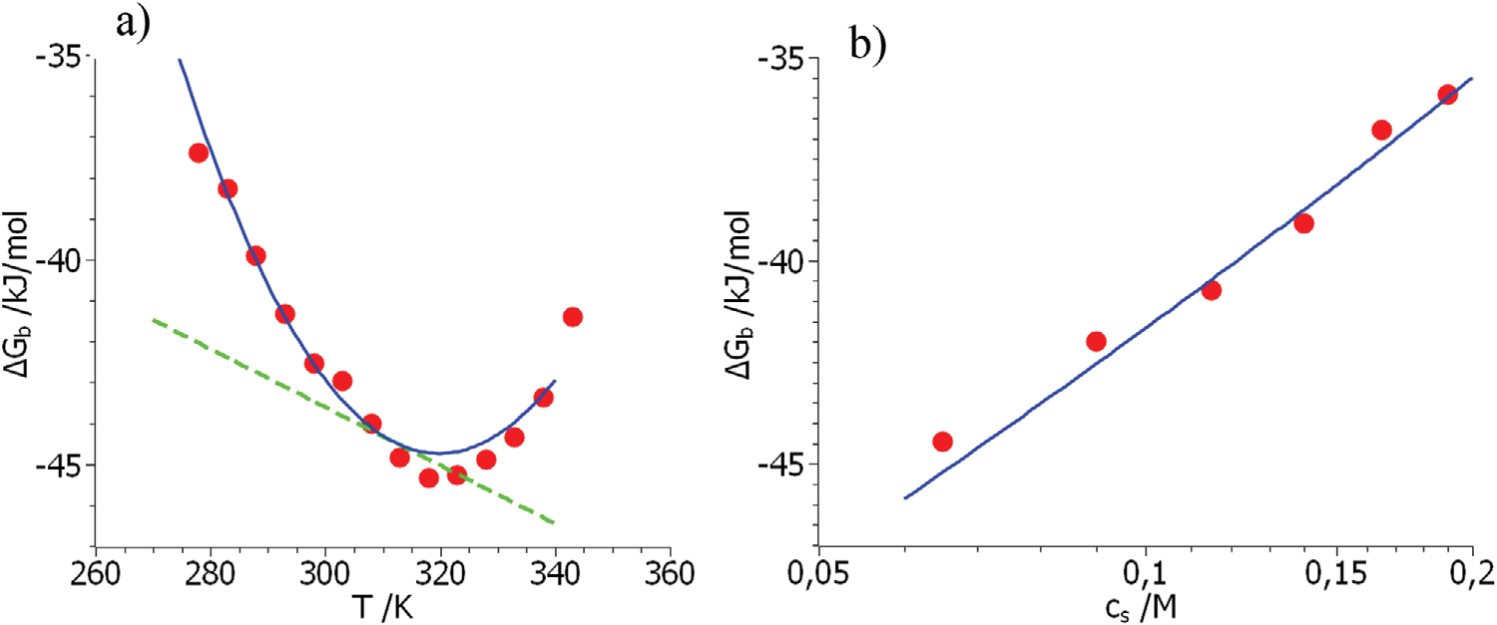
Comparison of the experimental data of Δ*G*_b_ (red points) obtained for the system Klentaq/DNA^[^ ^7^, ^8^ ^]^ with Equation (21). (a) Displays the dependence on temperature whereas (b) gives the respective comparison as the function of salt concentration measured at 298 K. The solid lines mark the fit by Equation (21) using the parameters gathered in Table 1. The dashed line in (a) shows the terms *RT*Δ*n*_ci_ln*c*_s_ + Δ*H*_0_ − *T*Δ*S*_0_ (see Equations (21) and (23)).

It is interesting to compare the present theory to data taken from systems in which DNA interacts with proteins. Here we use the data of Datta and LiCata on the interaction of DNA with the polymerase Klentaq.^[^ ^7^, ^8^ ^]^ **Figure** **3**a displays the free energies of binding taken at a salt concentration of 0.09 m as the function of temperature^[^ ^8^ ^]^ whereas Figure 3b shows Δ*G*_b_ obtained at a temperature of 298 K as the function of *c*_s_.^[^ ^7^ ^]^ It should be noted that the dependence of Δ*G*_b_ on temperature was only measured at a single salt concentration. Also, the dependence on *c*_s_ is only available for a singe temperature which may restrict the accuracy of the derived parameters. The points in Figure 3 mark the experimental data whereas theory is shown by a solid line. The parameters of the model have again been determined from a simultaneous fit of the entire set of data using the MathLab tool *cftool*. The respective parameters are again gathered in Table 1.

Figure 3a,b demonstrates that full agreement of theory and experiment can be reached: The dashed line display the terms *RT*Δ*n*_ci_ln*c*_s_ + Δ*H*_0_ − TΔ*S*_0_ (cf. Equation (21)) which are the leading terms in absence of any strong contribution of hydration. They give the entire free energy of binding Δ*G*_b_ when *T = T*_0_. However, if the temperature is lower or higher than *T*_0_, there are deviations caused by hydration. These deviations can be read off directly from the difference between the green dashed line (mainly counterion release) and the blue solid line giving the full free energy of binding. The changes are of the order of a few kJ mol^–1^ and lead to the characteristic parabolic shape of Δ*G*_b_ as the function of temperature. It is hence evident that Equation (21) can describe both the characteristic dependence of Δ*G*_b_ on temperature as well as on salt concentration in a fully quantitative manner. The skewed parabolic shape of Δ*G*_b_ when plotted against *T* originates from the linear decrease of the free energy due to counterion release onto which the approximately parabolic dependence on *T* due to the last term in Equation (21) adds up. This interplay of both effects, namely counterion release and hydration was already obvious from the discussion of Figure 2a. Figure 3b demonstrates that the dependence of Δ*G*_b_ on the concentration of added salt can be described as well. There is a slight curvature due to hydration which is hardly beyond the experimental error. Determination of Δ*n*_ci_ from a linear fit of these data may therefore lead to a small error of this parameter and to a spurious dependence on temperature. It is interesting to note that Equation (21) is fully compatible with Equation (22) with a term Δ*c*_p,0_ which is independent of concentration. The fit of the experimental data to Equation (21) leads to Δ*c*_p,0_ = 0 within experimental error. Hence, complex formation in the system DNA/Klentaq is fully described by counterion release in conjunction with water release as embodied in Equation (19).

As discussed previously, the residual free energy Δ*G*_res_ = Δ*H*_0_ − *T*_0_Δ*S*_0_ describes the free energy of the complex at direct contact.^[^ ^40^ ^]^ The present model allows us to split up this term into an enthalpic contribution Δ*H*_0_ and an entropic one Δ*S*_0_. The data gathered in Table 1 show that for all system under consideration here there is a marked enthalpic term that may originate from salt bridges or hydrogen bonds. The entropic term is more difficult to discuss inasmuch it may contain considerable contributions from changes in conformation during binding.

**Figure 4:**
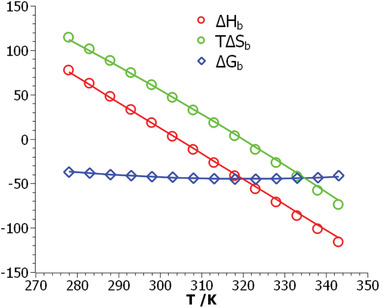
Comparison of thermodynamic data obtained for the system Klentaq/DNA.^[^ ^7^, ^8^ ^]^ The red circles display the binding enthalpies obtained by application of Equation (11) whereas the green circles show the respective entropies multiplied by *T*. The blue diamonds denote the free energies obtained experimentally from ITC. The solid lines mark the theoretical results: red line: binding enthalpy Δ*H*_b_ calculated from Equation (16), green line: *T*Δ*S*_b_ as obtained from Equation (20), and blue line: Δ*G*_b_ according to Equation (21). See also the discussion of Figure 3.

For systems with a maximum of Δ*G*_b_ in the experimental range of temperatures, Equation (12) is exact and allows us to obtain the binding enthalpy Δ*H*_b_ and the binding entropy Δ*S*_b_ directly from the measured free energy Δ*G*_b_. The wide range of temperature available for the system DNA/Klentaq provides a good basis for this decomposition. **Figure** **4** displays both Δ*H*_b_ and Δ*S*_b_ together with the theoretical results obtained by Equations (16) and (21), respectively. The open symbols present the data obtained through application of Equation (21) whereas the solid lines show the results of theory. Full agreement between theory and experiment is reached which furthermore corroborates the validity of the theoretical model. It demonstrates that the hydration term in Equation (21) is the origin of the strong enthalpy–entropy cancellation as expected.

## 4 Conclusion

We presented a simple phenomenological model for the free energy Δ*G*_b_ of the complex formation between a polyelectrolyte and a protein in aqueous solution. The model covers the leading factors for binding, namely counterion release and the release or uptake of water during complex formation. Counterion release is covered by the first term in Equation (21) whereas the effect of water release as introduced in Equation (5) determines the dependence of Δ*G*_b_ on temperature. The term Δ*w* interpreted so far as the number of released water molecules is related to the free energy of hydration and vanishes at a characteristic temperature *T*_0_. Theory is capable of describing the free energy Δ*G*_b_ on temperature as well as on salt concentration in a fully quantitative manner. Equation (21) also shows that hydration is the origin of the strong enthalpy–entropy cancellation. This is shown in Figure 4 which displays the free energy of binding Δ*G*_b_ together with the enthalpy of binding Δ*H*_b_ and the respective entropy of binding *T*Δ*S*_b_ obtained through application of Equation (12). The free energy of binding is nearly independent of temperature whereas enthalpy and entropy exhibit a marked dependence on *T*. This finding which is typical for many complexes of polyelectrolytes with proteins can be easily explained in terms of the present model. Therefore the present model allows us not only to model the interaction of polyelectrolytes with proteins but also to understand a long‐standing and controversial subject in the field.

The present work also demonstrates that studies of complex formation between a polyelectrolyte and a protein should comprise the investigation of the binding constant as the function of both *T* and *c*_s_. Moreover, these investigations should be done using salts that exhibit defined Hofmeister effects. A comparison with the SPM could then quantify the role of given ions for complex stability and compare this information to the rich literature on protein denaturation^[^ ^30^ ^]^ and also on work done on synthetic polymers.^[^ ^61^ ^]^

## Conflict of Interest

The authors declare no conflict of interest.

## Data Availability

Data are available on request from the authors.
